# The Potential of Selected Plants and Their Biologically Active Molecules in the Treatment of Depression and Anxiety Disorders

**DOI:** 10.3390/ijms26052368

**Published:** 2025-03-06

**Authors:** Nicol Urbanska, Tolulope Joshua Ashaolu, Simona Mattova, Patrik Simko, Terezia Kiskova

**Affiliations:** 1 Institute of Biology and Ecology, Faculty of Science, Pavol Jozef Safarik University in Kosice, Srobarova 2, 041 54 Kosice, Slovakia; 2 Institute for Global Health Innovations, Duy Tan University, 254 Nguyen Van Linh Street, Thanh-Khe District, Da Nang 550000, Vietnam; 3 Institute of Pathology, Faculty of Medicine, Pavol Jozef Safarik University in Kosice, Rastislavova 43, 040 01 Kosice, Slovakia

**Keywords:** depression, anxiety, herbal medicine, plants, biologically active substances

## Abstract

The incidence of anxiety and depression disorders is increasing worldwide. There is an increasing incidence of hard-to-treat depression with various aspects of origin. Almost 80% of people prefer to use natural remedies and supplements as their primary healthcare solution. Not surprisingly, around one-third of drugs were inspired by nature. Over the past three decades, the use of such remedies has increased significantly. Synthetic antidepressants may cause various negative side effects, whereas herbal medicines are favored because of their ability to relieve symptoms with minimal to no side effects and lower financial burden. This review provides an overview of herbs and biologically active compounds used to treat depression.

## 1. Introduction

Major depressive disorders (MDDs) were ranked as the 3rd cause of the disease burden in 2008, and it is projected that it will be the leading cause by 2030 [1]. The COVID-19 pandemic has increased the incidence of depression and mood disorders [2]. Depression is diagnosed when an individual experiences persistently low mood, poor concentration, changes in appetite, anhedonia, feelings of guilt or worthlessness, a lack of energy, sleep disturbances, or suicidal thoughts [3,4]. There are similarities in the neurobiological changes that cause depression and neurodegenerative diseases, such as Alzheimer’s, Parkinson’s, and Huntington’s diseases [5]. However, depression is often a comorbidity of other illnesses, including Alzheimer’s disease, epilepsy, Parkinson’s disease, migraine, and stroke [6,7].

Depression results from a combination of genetic and environmental factors; however, the exact pathology and cause of the disease are still not well understood [8,9,10]. Epidemiological studies have shown that genetics play a role in up to 80% of cases of depression and bipolar disorder [11]. Epidemiological studies of bipolar disorder show a heritability of 65–80%, while according to the Psychiatric Genomics Consortium and Genome-wide Association Study (GWAS), the heritability based on single-nucleotide polymorphisms is ~20%, indicating several genetic factors that need to be elucidated [12,13]. The highly polygenic architecture of genetic markers of bipolar disorder overlaps with MDD, schizophrenia, and other diseases [14].

Epigenetics plays a special role in depression through short- and long-term gene expression variations that are caused by non-DNA-encoded mechanisms, including DNA methylation or hydroxymethylation, histone modifications, expression of noncoding RNAs, chromatin remodeling, and RNA modification [15].

Another role in the origin and progress of depression is played by the gut microbiome. There is a communication network between the gut and the brain that is bidirectional, dynamic, and complex. Neuropsychiatric, neurodegenerative, or metabolic disorders are also caused by changes in the gut–brain axis. The regulator of this axis is the gut microbiota through metabolic, neuroendocrine pathways. Through histone modification and gene silencing associated with non-coding RNA and DNA methylation, the gut microbiome can modulate diseases. Short-chain fatty acids such as butyrate are also important inhibitors of histone deacetylases. Dysbiosis and epigenetics play an important role in the pathophysiology of depression [16]. The gut–brain axis may also be involved in the development of depression through the metabolism of tryptophan, which has been observed in the hippocampus [17]. The gut–brain axis also has a direct relationship with the HPA active in depression [16]. Depressed patients suffer from a reduced number of *Bifidobacterium* and *Lactobacillus* species in the intestinal microbiome, which can lead to inflammation. Probiotic treatment has been shown to be effective [11,18].

In addition to traditional antidepressants, the antidepressant ketamine is also coming to the fore, which accelerates the antidepressant effect of antidepressants by inhibiting the ionotropic glutamate receptor NMDA (N-methyl-D-aspartate) [19,20]. Other innovative therapies involve neurofeedback, a non-invasive intervention that has been increasingly used as a potential treatment for major depressive disorders [21], digital psychotherapy [22], or psychobiotics [23]. However, these approaches need to be validated more deeply and for a longer time.

Research in both preclinical and clinical fields points to several theories related to depression, including monoamines, receptors, the hypothalamus–pituitary axis (HPA), the endocrine system, inflammation, neuroplasticity, mitochondria, neurogenesis, growth factors, circadian rhythms, oxidative stress [24,25], and glutamate [26]. The monoamine hypothesis suggests that depression is caused by a deficiency in neurotransmitters, such as serotonin, dopamine, and noradrenaline [27]. Synthetic antidepressants have been used to treat depression and anxiety since the 1950s [28]. The disadvantages of using synthetic antidepressants include a delayed onset of action, with a time frame of 2–4 weeks, during which serious consequences can occur. Additionally, these drugs often have low efficacy and high toxicity and can cause negative interactions with other medications [24]. First-generation antidepressants, such as tricyclic and monoamine oxidase inhibitors, have largely been replaced by second-generation antidepressants, such as selective serotonin reuptake inhibitors (SSRIs) [29], which are considered safer but also have serious side effects, such as gastrointestinal issues, sexual dysfunction [30], emotional blunting, and weight gain [31,32], and can also increase the risk of suicidal tendencies in some children and teenagers [33]. Synthetic antidepressants have also been linked to various side effects in the treatment of anxiety and neurological diseases [34], and the discontinuation of their use can cause symptoms, such as balance disorders, anxiety, depression, and nausea. Despite the use of antidepressants, 50% of patients receiving monotherapy do not respond effectively, and 20–30% of patients do not respond to a specific antidepressant [35].

Depression is often accompanied by other psychiatric disorders, particularly anxiety, and, to a lesser extent, alcohol or drug abuse. The onset of anxiety usually occurs before depression, whereas the onset of alcohol abuse can occur either before or after depression. This high comorbidity rate highlights the complex nature of depression and the need for a comprehensive treatment approach [4].

Anxiety is caused by excessive stimulation of the neuronal circuit, where the hippocampus and amygdala play a key role in regulating emotions and emotional memory, along with the HPA and sympathetic systems [36,37]. The treatment of anxiety focuses on regulating neurotransmitters, such as serotonin and noradrenaline, as well as the amygdala nuclei, and their interaction with corticotrophin-releasing hormone (CRH) through neuroimaging techniques, such as magnetic resonance imaging (MRI) and tomography [38,39]. The underlying cause of anxiety is an imbalance in neurotransmitters, including γ-aminobutyric acid (GABA), serotonin, noradrenaline, and dopamine. Vigilance is regulated by the locus coeruleus, which is responsible for regulating noradrenaline and is thought to play a role in anxiety, panic, and fear. Forty percent of patients receiving treatment for anxiety also experience an MDD [38].

**Figure 1:**
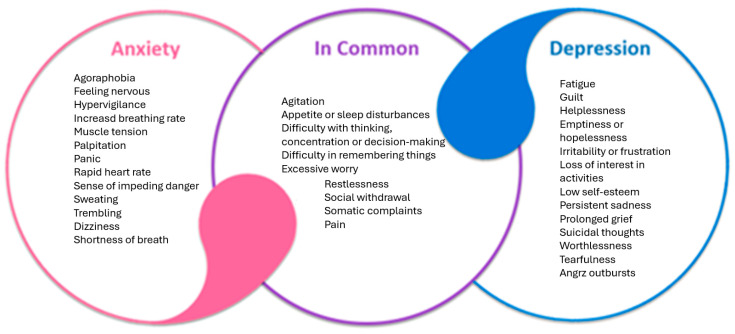
Common features of depressive and anxiety disorders.

Anxiety and depression are two of the most prevalent psychiatric disorders and often co-occur. In the late 1970s and the 1980s, the distinction between anxiety and depression began to blur, and it was acknowledged that these two disorders had overlapping characteristics (Figure 1). This is supported by biological evidence, such as the dysregulation of the HPA and brain response to serotonergic challenge, which are common in both anxiety and depression [40,41].

Selection criteria for review: Flavonoids are phenolic substances isolated from a wide range of vascular plants, with over 8000 individual compounds known. To date, more than 4000 different flavonoids have been identified from plant origin. The PubMed and Scopus databases were searched using keywords, such as depression, anxiety, and natural products. The internal criteria were set. We selected 10 plants with described antidepressant activity from the huge number, which may be a limitation of our study. From these, famous ones, such as hypericin or Ginko biloba, were selected, along with others not so well known for affecting depression. All the obtained articles were reviewed, and according to the inclusion and exit criteria, the related articles were selected to write this review article. The Google Scholar database was also searched for reliability.

## 2. Herbal Versus Modern Medicine

The World Health Organization (WHO) defines herbal medicine as a practice that includes herbs, herbal materials, preparations, and finished products that use parts of plants or other botanical materials as active ingredients [42]. Herbal medicine, also known as phytomedicine or botanical medicine, has a long history and utilizes parts of plants, such as roots, stems, leaves, berries, and flowers, for therapeutic purposes [43]. The WHO states that medicinal plants contain substances that can be used for various therapeutic purposes and serve as precursors for semi-synthesis of chemo-pharmaceuticals [44]. Plant drugs include not only the active components found in plant parts (phytochemicals) but also raw or processed plant materials with added ingredients, such as preservatives, solvents, or dilutions. Over time, some phytochemicals have been shown to provide health benefits to humans [45]. The use of herbal medicines dates back 60,000 years [46], as recorded in Babylon, and each region has its own unique healing practices, philosophies, and systems, such as traditional Chinese medicine [45]; Ayurveda, which is recognized by the Indian government; and Kampo medicine in Japan [47,48]. The WHO defines traditional medicine as a combination of knowledge, skills, and practices based on the beliefs and experiences of various cultures that are used to maintain health and treat physical and mental illnesses [49]. Despite extensive documentation of medicinal plants, their use in culture lacks an understanding of their constituents or function [50].

The WHO has issued guidelines for the proper evaluation of herbal medicines, including the assessment of their quality, stability, safety, and effectiveness. The Food and Drug Administration (FDA) also follows these guidelines to verify plant-based products [45]. However, a safety assessment of herbal medicines can be complex because of the presence of multiple phytochemicals in medicinal plants [51].

On the other hand, the era of modern medicine began at the beginning of the 19th century [52]. Of all pharmaceutical products, 25% are herbal medicines, such as picrotoxin and aspirin [53]. The first drug isolated from plant sources was morphine, a benzylisoquinoline alkaloid derived from the opium poppy (*Papaver somniferum*), which was approved in 1827 as a pain-reliever [54]. The main advantage of conventional medicine is that the proper dosage is ensured, which is crucial because the dose of a drug determines whether it will have optimal effectiveness, cause toxicity, or have no effect [55]. The approval of a new drug is based on preclinical and clinical studies that provide information on the dose and administration route that produces therapeutic effects without adverse reactions [56]. Precise molecular pathways can be defined for a single molecule in a pill.

## 3. Herbal Medicine and Herbal Extracts in Depression

It is essential for today’s society to have more effective and less toxic antidepressants with fewer negative interactions with other drugs for the treatment of neurological, psychiatric, and neurodegenerative diseases [57,58,59]. Natural antidepressants may offer a solution to the limitations of synthetic antidepressants [60].

Many people worldwide, especially in developing countries, rely on alternative natural bioactive substances to treat depression because of their potential efficacy and lower financial burden compared to synthetic antidepressants [61,62]. In the past three decades, the use of natural medicines and supplements has increased, with 80% of the population using them as a primary form of healthcare [63,64]. Numerous plant species have been found to be efficacious in treating depression and central nervous system (CNS) diseases [65,66].

Plants contain natural components such as primary and secondary metabolites or other biomacromolecules, each of which plays a specific role in the organism [67]. Pharmacognosy studies the properties of plant-derived substances to discover new medicines, such as aspirin from willow bark [68]. However, the interactions between biologically active substances in plants are complex and influence the overall biological effects of the extract [69]. The idea that a single substance can treat a disease, known as the “silver bullet”, is now considered inadequate in clinical practice [70]. Herbal medicine is often referred to as the “herbal shotgun” because of the presence of many compounds in the extract. Multifactorial diseases are typically treated with a combination of drugs known as polypharmacy [69,70,71]. However, a recent study showed that polypharmacy leads to more adverse drug reactions and does not offer any benefits over monotherapies [72].

Biological effects can also be achieved through polyvalency, in which multiple components work together to produce a therapeutic effect. The multicomponent approach in herbal medicine creates alternative products with multiple targets and minimal side effects, making them suitable candidates for the treatment of CNS diseases [73]. Plants with favorable effects on the nervous system are, for example, genus *Gladiolus* [62], *Hypericum* [74], *Crocus sativus* [75,76], *Aloysia* [77], *Hemerocallis* [78], *Allium* [79], *Piper methysticum* [80], *Lavandula officinalis* [81,82,83], *Rhodiola rosea* [84], *Ginkgo biloba* [85,86], *Salvia officinalis* [87,88], *Cannabis sativa* [89], *Convolvulus pluricaulis* [90], *Echinacea angustifolia* [91], and many other plants, such as those used in Chinese medicine [92].

It has been shown that bioactive substances found in plants can have neuroprotective effects in the treatment of mental health conditions, such as depression and neurodegenerative diseases. These substances work through a variety of mechanisms, including the modulation of signaling pathways through different receptors, enzymes, and proteins [6,93,94]. Plant metabolites have been found to bind to neurotransmitter receptors and thus alter the synthesis and function of neurotransmitters [95]; regulate the CNS and endocrine system [96,97,98]; and exert anxiolytic, hypnotic, antidepressant, nootropic, sedative, analgesic [97], tonic, and adaptogenic effects [99]. The interconnected nature of the mechanisms of action in herbal medicine can also help to treat comorbidities, such as resolving anxiety when treating depression or alleviating depression when treating insomnia [100,101]. The effectiveness of herbal medicine is still being determined through “omics” technologies, such as pharmacogenomics, metabolomics, proteomics, and epigenetics [102,103].

**Figure 2:**
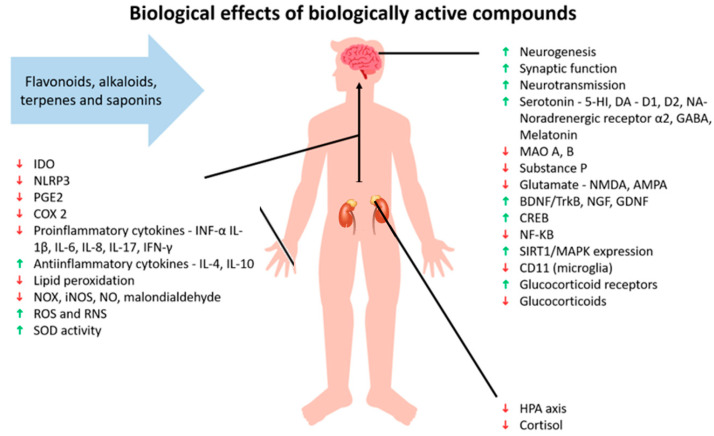
Effects of biologically active compounds. Abbreviations: indoleam-ine-2,3-dioxygenase (IDO); NLR family pyrin domain containing 3 (NLRP3); prostaglandin E2 (PGE2); cyclooxygenase-2 (COX 2); tumor necrosis factor α (TNF-α); interleukin (IL); oxides of nitrogen (NOX); inducible nitric oxide synthase (iNOS); nitric oxide (NO); reactive oxygen species (ROS); reactive nitrogen species (RNS); superoxide dismutase (SOD); 5-hydroxytryptamine receptor (5-HT); dopamine (DA); dopamine receptor D1; dopamine receptor D2; noradrenaline (NA); γ-aminobutyric acid (GABA); monoamine oxidase (MAO); N-methyl-D-aspartate (NMDA); α-Amino-3-hydroxy-5-methyl-4-isoxazolepropionic acid (AMPA); brain-derived neurotrophic factor/tropomyosin receptor kinase B (BDNF/TrkB); nerve growth factor (NGF); glial cell line-derived neurotrophic factor (GDNF); cAMP-response element-binding protein (CREB); nuclear factor kappa B (NF-κβ); sirtuin 1/mitogen-activated protein kinase (SIRT1/MAPK); CD11b (microglia marker); and hypothalamus-pituitary axis (HPA). Green arrow indicates support or increase of the individual matter, red arrow indicates decline or decrease of the individual matter.

Herbomics involves the use of advanced technologies to study the effects of plants in phytotherapy and can provide clues about the toxicity and effects of given plants [101]. An example is a study that investigated the regulation of gene expression in the hypothalamus of male rats after treatment with imipramine (a tricyclic antidepressant) and *Hypericum perforatum* for eight weeks. The results showed that both treatments affected genes related to energy production/expenditure, cell structure, neurotransmission, fatty acid metabolism, protein synthesis/degradation, hormones, ion concentration, repetitive DNA sequences, transcription regulation, signal transduction, and synaptic transmission [104] (Figure 2). Another study found that *Hypericum perforatum*, clomipramine (a synthetic antidepressant), and a mixture of herbs called Xiao-yao-san changed the expression of different proteins that were mainly involved in energy metabolism. Both clomipramine and *Hypericum perforatum* were found to have similar gene expressions, as demonstrated by the increased expression of two forms of dihydropyrimidinase-related protein 2, which are involved in regeneration, the outgrowth of axons, and heat shock protein (Hsp-70), a neuronal protein-folding protein [105].

Antidepressant effects of different plants have been demonstrated. Among them, *Hypericum perforatum* is a plant native to Asia and Europe [74,106] and is an invasive plant in North and South America, Australia, and South Africa [107]. Among the genus *Hypericum*, which also includes *Hypericum perforatum*, there are 450 species [108]. The plant is used in traditional herbal medicines, such as Greek medicine, traditional Chinese medicine, and Islamic traditional medicine [109], for the treatment of many various disorders [109,110,111]. It is used in the form of capsules, tea, tablets, or liquid extract [112].

The main biologically active substances responsible for the antidepressant effect are hyperforin (derivative of phloroglucinol), hypericin (naphthodianthrone) [113], and pseudohypericin (naphthodianthrone) [108]; however, the presence of rutin (a flavonoid) also plays a role in its antidepressant effects [114], as well as quercetin, kaempferol, leteolin [115], quercitrin, or hypeoside [108]. These substances can also prevent the relapse of depression [116]. The therapeutic effect of the plant has been investigated in studies not only with depression [117] but also with anxiety [118], and its anti-inflammatory, antinociceptive, healing effect (wounds) has also been observed [108]. The biologically active substances of *Hypericum perforatum* influence the levels of neurotransmitters such as norepinephrine, dopamine, and serotonin by the non-selective inhibition of their reabsorption, increasing the levels of 5-hydroxytryptamine 1A receptor (5-HT1A), 5-hydroxytryptamine 1B receptor (5-HT1B), and 5-hydroxytryptamine receptor 2 (5-HT2) and selectively inhibiting monoamine oxidase A (MAO A), monoamine oxidase B (MAO B), glutamate, dopamine, and β receptors in the prefrontal cortex [119,120]. In addition to the neurotransmitters mentioned, GABA and NMDA are affected [121,122,123]. During chronic stress, *Hypericum perforatum* had an antidepressant effect through the reuptake of serotonin, noradrenaline, and dopamine [124]. An extract of *Hypericum perforatum* also negatively affected the neuronal uptake of GABA and L-glutamate [125]. In vitro studies have shown that *Hypericum perforatum* inhibited the binding of [3H]flumazenil to benzodiazepine binding sites of γ-aminobutyric acid type A receptors (GABA-A) in a rat brain [126]. In rats, an increase in acetylcholine levels in the hippocampus and transcription of cyclic AMP-responsive element-binding protein (CREB), decarboxylase (GAD) in the bed nucleus of the stria terminalis, and proopiomelanocortin (POMC) in the pituitary gland were found [127]. In vitro studies with HT-22 cells have shown that *Hypericum perforatum* reduces lipopolysaccharide-induced inflammation and increases neuronal growth [128]. The extract from *Hypericum perforatum* also had an effect on the decrease in corticosterone and adrenocorticotropic hormone (ACTH) [129]. A recent study according to Song et al. (2022) reported an antidepressant effect of the aqueous extract of *Hypericum perforatum* during chronic unpredictable mild stress (CUMS) in rats and mice via the NLR family pyrin domain containing 1 (NLRP1) inflammasome and the CXC motif chemokine ligand/CXC motif chemokine receptor 2/brain-derived neurotrophic factor (CXCL/CXCR2/BDNF) signaling pathway [130]. Clinical studies have found the effect of *Hypericum perforatum* to be comparable to that of SSRIs and tricyclic antidepressants [131] but better tolerated [132]. However, the combination of *Hypericum perforatum* and synthetic antidepressants may reduce their efficacy by increasing cytochrome p450 activity [119,133]. Drug interactions with *Hypericum perforatum* via the modulation of cytochrome p450 may cause adverse effects, such as gastrointestinal symptoms, headache, dizziness, anxiety, fatigue, mania, confusion, neuropathy, alopecia, hypersensitivity, and sedation [108].

The herb has not been approved by the FDA and is considered a dietary supplement [134], even despite the fact that it has proven antidepressant effects in studies [122]. Some of the mechanisms of *Hypericum perforatum* are found in approved medications for the same complications [127]. Remotiv is a demonstrably commercial product containing *Hypericum perforatum* extract (referred to as Ze 117), which alleviates stress, nervous tension, and depression and balances emotional stability. The effectiveness of the product has been investigated in preclinical [135,136] and clinical studies [137,138]. Remotiv dry extract tablets at a dose of 250 mg improved short-term spatial and verbal memory in healthy volunteers. The nootropic effect was not observed at the 500 mg dose. Improvement in emotional balance and mood was observed at both doses. The mechanism of action of Remotiv may involve the enhancement of dopaminergic transmission [138]. The modulation of neurotransmitters such as serotonin, dopamine, and noradrenaline has been demonstrated in preclinical studies. Remotiv, when used long term, increases the concentration of neurotransmitters in the synaptic cleft by reducing the presynaptic reuptake of neurotransmitters [139,140,141,142]. One study suggests that Remotiv may reduce alcohol intake and thus may be helpful in the treatment of alcoholism [143]. The relationship of alcohol abuse with depression and anxiety is common [144]. In the treatment of mild-to-moderate MDD, in a systematic review that looked at 35 studies with the efficacy of *Hypericum perforatum* over a period of 4–12 weeks, the efficacy of *Hypericum perforatum* was higher than the placebo and similar to conventional antidepressants [145].

*Rhodiola rosea* (“golden root”) was located and identified by G.V. Krylov in 1961 during the expedition to the Altai Mountains in southern Siberia [146]. *Rhodiola rosea* is a plant used in traditional medicine in Eastern Europe and Asia [147,148] and in Russia [149]. In traditional medicine, it has been used to increase work productivity; reduce fatigue and impotence; resist symptoms of altitude sickness, anemia, infections, and gastrointestinal diseases; increase fertility; and address influenza, tuberculosis, cancer, scurvy, hemorrhoids, headache, and especially depression [149,150]. *Rhodiola rosea* contains up to 140 compounds found in underground parts of the plant [149]. It contains components such as herbacetin, rhodiosin belonging to flavonoids, geraniol (monoterpene), tyrosol, salidroside, phenylethanol derivatives belonging to triterpenes, and also rosarin, rosin, rosdirin, and rosavin belonging to phenylpropanoid glycosides [147,148]. When comparing *Rhodiola rosea* components, a stronger antidepressant effect in in vivo depression models at a dose of 0.26 mg/kg was achieved by rhodioloside/saliroside compared to five components—rosavin, rosin, tyrosol rhodioloside/saliroside, and rosarin. However, the strongest effect occurred with the combination of ingredients, which indicates a synergistic effect. Depending on the dose of 10, 20, or 50 mg of *Rhodiola rosea* extract in vivo in the forced swim test (FST), there was an increase in swimming time, which demonstrated an antidepressant effect and was higher compared to the administration of *Hypericum perforatum* and imipramine [151]. Studies in mice have shown that *Rhodiola rosea* extract can reduce depression-like behavior and increase exploratory behavior in models of CUMS [152]. Plant extracts at a dose of 1.5–6 g/kg have been shown to increase neurogenesis and the proliferation of stem cells in the hippocampus of rats [153]. It also affects the release of various neuropeptides and decreases HPA and G protein-coupled receptor (GPCR) activity [154]. The active ingredient, salidroside, has antidepressant effects in rats, possibly related to the regulation of inflammation and the HPA [155]. In addition, it has anti-inflammatory and neuroprotective effects [156].

*Rhodiola rosea* is considered a dietary supplement [157] and an adaptogen [158]. In the prevention and treatment of depression, *Rhodiola rosea* has shown efficacy [159]. The effects of *Rhodiola rosea* in the treatment of cognitive and psychiatric disorders are the same as those of conventional drugs. However, it does not lead to addiction and does not exceed the body’s ability to respond to and defend against stress, and it also does not prevent the body from returning to a functional state after the drug’s effect has worn off [87]. According to Yu et al. (2019), *Rhodiola rosea* alleviates depression and anxiety in patients through lipid peroxidation in the mitochondrial respiratory chain and the inhibition of reactive oxygen species (ROS) [160]. Clinical studies have also shown that it can improve mild-to-moderate depression without side effects; modulate neurotransmitters such as norepinephrine, serotonin, and dopamine; and decrease MAO A and MAO B activity [161,162]. In addition to these effects, an interesting finding from one study was that *Rhodiola rosea* extract at a dose of 500 mg reduced LTD (long-term depression) compared to the placebo, yet it did not affect cortical excitability [163]. Over 6 weeks, the extract from *Rhodiola rosea* in a dose of 340–680 mg in adult patients with mild and moderate depression had a beneficial effect [164]. In patients with mild anxiety, after 14 days of administration of 200 mg of *Rhodiola rosea* two times a day, anxiety, stress, depression, and anger were reduced compared to the placebo [165]. *Rhodiola rosea* does not show toxicity [157,166]. No side effects attributed to the effects of *Rhodiola rosea* have been reported in clinical studies [167]. In humans, a dose of 680 mg per os of *Rhodiola rosea* does not cause any serious side effects [164]. Also, in animal studies, *Rhodiola rosea* did not show acute or chronic toxicity [166,168]. With the exception of an older study in rats at a dose of 3.360 g/kg, it showed low toxicity [167]. *Rhodiola rosea* extract has shown an antidepressant effect in adults compared to a conventional antidepressant. *Rhodiola rosea* extract was well tolerated during short-term administration; however, it had no significant effect on mild anxiety [165]. The combined treatment of rhodiola and saffron was studied for mild depression. Oral administration of 154 mg rhodiola and 15 mg saffron twice daily for 6 weeks showed improvement in depression and anxiety in 45 adults [169].

In Chinese medicine, *Ginko biloba* has been used for more than 5000 years to treat cough, enuresis, and asthma [170]. In addition to terpenoids (diterpenes ginkgolides A, B, C, J, M, N, K, and L) and flavonoids (aromadendrin and 5,7,4 trihydroxy-flavones), *Ginkgo biloba* also contains polyprenols (di-trans-poly-cis-octadecaprenol), organic acids (ginkgolic acid), and also groups of flavones, flavanol glycosides, and aglycones, such as quercetin, rutin, kaempferol, apigenin, isorhamnetin, myricetin, and luteolin. Terpenoids protect against mitochondrial damage, which may act through two mechanisms: first, through platelet-activating factor (1-O-alkyl-2-acetyl-sn-glycero-3-phosphocholine) receptor antagonism, as platelet-activating factor (PAF) antagonists reduce neuronal damage and act preventively against ischemia; second, through the interaction of chloride channels [170], as terpenoids block the chloride channel in hippocampal neurons in rats [171]. In addition, terpenoids have vasoregulatory and antioxidant activity, free radical scavenging, and neuroprotective and protective effects against memory and learning disorders. Flavonoids also prevent the formation of ROS and change the expression of antioxidants [170]. Even a higher effect in suppression of free radicals was demonstrated compared to terpenoids [172]. Through the interaction of neuronal receptors, they influence the processes of learning, memory, and cognitive functions, as they cause cerebrovascular blood flow and synaptic plasticity [170]. In addition, they reduce proinflammatory cytokines, such as TNF-α, prostaglandin E2, IL-1β, and NF-kB [173]. Ginkgolic acids, the phenolic compounds isolated from the leaves and seeds of *Ginkgo biloba*, can cause genotoxic, cytotoxic, mutagenic, and carcinogenic effects [174]. For this reason, they are present in commercial preparations in amounts of <5 ppm. *Ginkgo biloba* has a beneficial effect on visual acuity, vertigo, atherosclerosis, tinnitus, and psychological and neurological disorders [170]. In addition, it has been shown in vitro that *Ginkgo biloba* protects neurons from degeneration during ischemic events [175]. *Ginkgo biloba* has demonstrated antidepressant effects in LH models in rats, where a dose of 150 mg/kg with 24% flavonoids and 6% terpenoids was found to prevent the corticosterone stress response (when administered two weeks before the test), although it did not improve the acquisition of active avoidance [176]. The reduction in depression-like behavior may be due to a reduction in MAO B, NO, and GABA [177].

The commercially available *Ginkgo biloba* extract EGb-761 balances monoamine activity, which is involved in anxiety and mood disorders [178]. The application of the EGb-761 extract increased the levels of serotonin and 5-hydroxyindoleacetic acid (associated with spatial memory) [179]. Although short-term administration of the EGb-761 extract did not affect serotonin levels, it increased noradrenaline and dopamine in the PFC [180]. In vitro, MAO inhibition was demonstrated by the EGb-761 extract [178] and also an increase in dopamine, noradrenaline, and acetylcholine neurotransmission [181]. The ginkgolide B component of the EGb-761 extract has an antidepressant effect, as it suppresses the increase in corticosteroids through the influence of benzodiazepine receptors [182]. The component ginkgolide B inhibited CRH and arginine vasopressin after per os application to rats for 14 days [183]. The in vitro extract EGb-761 increased testosterone and suppressed prolactin, which increases sexual behavior [184], which is often altered in psychiatric diseases [170]. BDNF is increased after the application of extract EGb-761 [185]. *Ginko biloba* leaf extracts are used as tablets, oral liquids, or injectable solutions [170]. Orally administered, EGb-761 extract is absorbed in the intestine and metabolized by the flora according to a pharmacokinetic study [186]. The data from the pharmacokinetics revealed terpene trilactones and flavonoids from *Ginkgo biloba* in the blood and in the brain, indicating that they may cross the blood–brain barrier [173]. *Ginkgo biloba* is approved in Germany for the treatment of dementia and used in the USA for the treatment of Alzheimer’s disease and dementia [170]. However, based on insufficient and inconsistent evidence, the FDA has not approved the *Ginkgo biloba* EGb-761 extract preparation for medical use, only as a supplement food product [187].

*Crocus sativus*, also known as saffron, is grown in hot and dry areas, such as Iran, Tibet, China, and India [188]. It has traditionally been used in Persian medicine to treat depression [189]. In clinical studies, 30 mg/kg of *Crocus sativus* was found to be equally effective in treating mild-to-moderate depression compared to 20 mg/kg fluoxetine and resulted in a reduction in depressive symptoms after 6 weeks, similar to the effects of 100 mg/kg imipramine and 40 mg/day citalopram (vs. 30 mg/day of *Crocus sativus*) [190,191,192]. The administration of 50 mg *Crocus sativus* twice daily for 12 weeks also decreased depression and anxiety [193]. A 6-week clinical study showed that *Crocus sativus* reduced symptoms of generalized anxiety disorder, whereas sertraline was not effective [194]. The active ingredients responsible for the antidepressant and anxiolytic effects of *Crocus sativus* include safranal [195] and crocin [196]. Safranal (an aldehyde organic compound and monoterpene belonging to essential oils) and crocin (a carotenoid and monoterpene belonging to essential oils) are involved in modulating neurotransmitters, such as glutamate, GABA, dopamine, noradrenaline, and serotonin [195,197,198,199,200]. Crocin inhibits the activities of cytochrome p450 family 2B (CYP2B), cytochrome p450 family 3A (CYP3A), cytochrome p450 family 2A (CYP2A), and cytochrome p450 family 2 C11 (CYP2C11), whereas safranal increases the activities of CYP3A, CYP2B, and CYP2C1 [201]. As reviewed, clinical studies have evaluated doses ranging from 20 to 400 mg/day of pure saffron. Dosages of up to 1.5 g/day of saffron are thought to be safe; toxic effects have been reported for 5 g doses. However, for mild-to-moderate depression, up to 30 mg/day of saffron extract (stigma or petal) is used [202].

*Melissa officinalis*, commonly used in Europe for treating the nervous system, has been found to exhibit antidepressant effects. This plant contains flavonoids, which are believed to be responsible for its antidepressant properties. Studies have shown that *Melissa officinalis* decreases the γ-aminobutyric acid transaminase (GABA-T) levels in the hippocampus [203]. In a double-blind randomized pilot study, *Mellisa officinalis* (2 g daily) showed a similar effect to fluoxetine in mild-to-moderate depression [204]. It ameliorated the depressive-like behavior of rats in a forced swim test via regulating the serotonergic neurotransmitters [22]. In another study, *Mellisa* was administered as supplementation for patients with depression, anxiety, and stress disorders. A total of 80 patients were included in the study. Eight-week supplementation with *Mellisa* capsules (3 g daily) resulted in decreased depression, anxiety, and stress-related symptoms [205]. The current evidence suggests that lemon balm (*Mellisa officinalis*) may be effective in improving anxiety and depressive symptoms, particularly in the acute setting [21]. The existing research indicates that *Mellisa officinalis* holds promise as a calming agent exhibiting both anxiolytic and antidepressant properties and can elicit cognitive and sleep-quality enhancement [23].

Flavonoids, unsaturated sterols, and saponins are responsible for the antidepressant effect of *Echium amoenum* occurring in Europe and the northern part of Iran [206,207]. The effect of flavonoids is comparable to that of SSRIs and tricyclic antidepressants (fluoxetine and imipramine) [208] and is more effective and better tolerated than SSRIs (citalopram) after 8 weeks [209]. Clinical trials and animal models have shown antidepressant, anti-inflammatory, analgesic, and antioxidant effects of *Echium amoenum* [210,211,212]. After 2 weeks, serotonin and dopamine levels in the cerebrospinal fluid were increased after oral administration of 125 mg/kg *Echium amoenum* in reserpine-induced depression in male Wistar rats [210]. Fifteen days after the administration of a dose of 5 mg/kg, *Echium amoenum* improved depression-like behavior (induced by Mn^2+^) in the FST, immobility time, body weight gain, and sucrose preference and reduced ROS, neurotoxicity, and apoptosis in the hippocampus [213]. In animal studies, the administration of *Echium amoenum* has been found to increase serotonin and dopamine levels, improve depression-like behavior, and reduce oxidative stress and neurotoxicity in the hippocampus [214,215,216,217].

*Curcuma longa*, also known as turmeric, is used as an antidepressant in China and India. Clinical trials have shown that curcumin, a component of *Curcuma longa*, reduced depression symptoms over 8 weeks [218]. Turmeric increases dopamine, BDNF, and serotonin levels [219]; inhibits the NLR family pyrin domain containing 3 (NLRP3), interleukin-1 beta (IL-1β), and nuclear factor kappa-light-chain-enhancer (NF-κB) of activated B cells, MAO A, and MAO B [220]; and causes a decrease in cortisol levels in saliva [221]. In a rodent model of depression, curcumin prevented memory loss, reduced anhedonia, improved abnormal levels of BDNF and extracellular-signal-regulated kinase (ERK), and reduced (ameliorated) autonomic activity [222]. In the FST and elevated plus maze (EPM) tests, curcumin showed antidepressant effects [223].

*Humulus lupulus* is commonly known as hops and has been found to improve symptoms of depression and anxiety in young people after four weeks of use [224]. This is believed to be due to the presence of various components such as humulone, xanthohumol, resveratrol, and others, which exert anxiolytic effects via the GABA receptor [216]. Hops have also been found to inhibit various P450 enzymes, such as CYP2C9, CYP1A2, CYP2C19, and CYP2C8 [215].

Lichen secondary metabolites and their biological properties have gained scientific attention from the beginning of the 20th century [225]. There are more than 1000 known lichen secondary metabolites so far [226]. All the secondary metabolites of lichens are of fungal origin in the form of crystals on the surface of hyphae and are not adequately soluble in water and are usually isolated from lichens using organic solvents [227]. The most studied lichen secondary metabolites include usnic acid, atranorin (ATR), or gyrophoric acid (GA). GA, as the most abundant lichen secondary metabolite in the *Umbilicaria families* [228,229], showed significant anxiolytic activity in an EPM test in laboratory animals with depression-like behavior when compared to untreated animals. Moreover, the neurogenesis level was restored to the level of healthy animals [230]. ATR, a dominant lichen secondary metabolite mainly in the families of *Cladoniaceae* [231], also showed significant anxiolytic activity in the EPM, as it was able to elevate the number of rearings and prolong the time spent in the open arms of the EPM test in treated animals when compared to the untreated depression-like group. The neurogenesis level as well as the level of mature neurons was increased [232].

Many other plants (such as many from Chinese medicine) have shown antidepressant effects; however, because of the limitations of this manuscript, only a select few have been described.

## 4. Biologically Active Substances with Antidepressant Effect

Plant metabolites with antidepressant effects have diverse chemical structures, including isoprenoids (terpenes), phenolic compounds, and alkaloids [233].

### 4.1. Flavonoids

Flavonoids (Table 1), a subgroup of secondary metabolites described by the diphenylpropane structure, are found in plants, fruits, and vegetables and have been shown to have health benefits, including the prevention of various diseases [234,235] and treatment of depression [236,237,238]. Specifically, flavonoid compounds are highlighted as robust defenders, addressing oxidative stress and inflammation to avert chronic illnesses [225]. Flavonoids include different classes, such as flavones, anthocyanins, flavonols, flavanols, catechins, flavanones, flavanonols, and chalcones [239]. Unsubstituted flavones and flavanones display the strongest antifungal activity; however, this activity generally decreases with the addition of methyl or hydroxyl groups to the flavone structure, though there are exceptions [226]. Anthocyanins, such as cyanidin-3-O-glucoside chloride, were shown to prevent the development and progression of urethane-induced lung cancer by regulating energy metabolism in mice [227]. The most famous catechin, epigallocatechin gallate (EGCG), may alleviate depression through interactions with gut microbiota and other mechanisms [229]. Moreover, the potential benefits of flavonoids for depression may be attributed to their antioxidant properties [240,241].

St John’s wort is a popular herbal remedy recommended by traditional Chinese medicine (TCM) practitioners and is licensed and widely prescribed for depression in many European countries up to now [228]. For patients with mild-to-moderate depression, St John’s wort has comparable efficacy and safety when compared with SSRIs [74], based on filtered data of FDA-approved and non-approved investigational antidepressive agents used in trials studying major depression [232]. During the analysis of investigational antidepressive agents, several drugs emerged as the most common candidates for study. Among the investigational drugs, several reached the highest phase, phase 4. These drugs include buprenorphine, reboxetine, tianeptine, mianserin, hypericin, and melitracen [232]. Hypericin is an excellent lead molecule since it differs structurally and mechanistically from all known antidepressants. There are hundreds of scientific publications dealing with the effects of hypericin during depression. The molecular mechanisms of action of hypericin include increasing presynaptic efficiency in the hippocampus [43], repairing the dysfunction of gap junctions in depression [242], neuroinflammation attenuation via the NLRP3 pathway [243], and many others.

As a counterpoint to hypericin, myricetin was selected to include in this publication. Myricetin has various pharmacological effects, including antioxidant, antiapoptotic, anti-photoaging, anticancer, antidiabetic, and anti-inflammatory effects [244]. Chronic administration of myricetin 50 mg/kg daily (i.p. 1 h before stress) for 21 days reduced immobility time in the FST and TST, thereby showing reducing depressive-like behavior, improved glutathione peroxidase activity in mice exposed to chronic stress, reduced plasma corticosterone, and normalized BDNF levels in the hippocampus, which may indicate an antioxidant effect [245]. In another study, albino mice and albino rats administered myricetin from *Vitis vinifera* Linn. at a dose of 10, 30, or 100 mg/kg showed anxiolytic effects in the EPM, open field test (OFT), light/dark apparatus test, and hole board apparatus test and after the administration of lithium 200 mg/kg and meta-chlorophenyl piperazine (MCP). Individual doses of myricetin were administered per os 1 h before the individual tests. Lithium was i.p. 1 h after myricetin administration and MCP 30 min before myricetin administration [246]. Myricetin increased neuronal proliferation, growth, and their survival, especially in the SVZ and SGZ [247]. In the last 5 years, myricetin was studied more for brain injury [248], post-ischemic neurodegeneration [249], or epilepsy [250], rather than depression. One study revealed that myricetin inhibited fear and anxiety-like behavior by HPA regulation and activation of the BDNF-ERK signaling pathway in posttraumatic stress disorder in Sprague-Dawley rats [230].

### 4.2. Alkaloids

The main sources of alkaloids are plants from the *Papaveraceae*, *Ranunculaceae*, *Amaryllidaceae*, and *Solanaceae* families [251,252]. These alkaloids are used to treat neurodegenerative diseases and include isoquinoline, oxindole, indole, pyrroindole, aporphine, piperidine, vinca, pyridine, β-carboline, and methylxantheme derivatives [253,254]. Indole, pyridine, and aporphine are agonists of muscarinic and adenosine receptors and antagonists of dopamine, nicotine, and NMDA, respectively. They also inhibit acetylcholinesterase, butyrylcholinesterase, and monooxygenase [253].

Indoles are a class of compounds that consist of carbon-nitrogen rings. They are found in various substances, such as mushroom alkaloids (psilocybin and ergine) and the drug lysergic acid diethylamide, and can affect serotonin activity in the brain [255].

**Table 1:** Selected biologically active compounds with antidepressant effects.

	Compound	Dose	Model	Activity	Ref.
flavonoids	hypericin	0.1–1 µM	ex vivo hippocampi from newborn Sprague-Dawley rats	enhancing presynaptic efficiency	[55]
		0.2 mg/kg	male Sprague-Dawley rats	increase in 5-HT levels in hypothalamus decrease in norepinephrine in hippocampus	[256]
		various	3808 human patients	comparable response to SSRIs	[74]
	rhodiosin	2 × 200 mg	50 human adults	improvement in mental speed improvement in mental resources	[257]
		340 mg	57 human patients	less antidepressant effect versus sertraline (50 mg) fewer adverse effects, well tolerated	[162]
	myricetin	50 mg/kg	male C57BL/6 mice	reduction in the immobility time in FST and TST normalization of BDNF levels improvement in glutathione peroxidase activity reduction in corticosterone in plasma	[245]
		10, 30, 100 mg/kg	albino mice and albino rats (strains not listed)	increase in time spent in open arms, number of entries in open arms in EPM increase in rearing in OFT increase in time spent in the lit area, number of transitions in light/dark apparatus increase in head poking in hole board apparatus reduction in lithium-induced head twitches reversal of MCP-induced anxiety	[246]
	luteolin	10, 30, 40 mg/kg	SPF male mice	ameliorating depressive-like behaviors promoting the Arg-1+ microglial phenotype reducing microglial proinflammatory responses reversing microglial phagocytosis-mediated synapse loss	[258]
terpenes	hyperforin	2.5, 5 mg/kg	male C57BL/6 J mice	reversed behavior in TST, FST, and splash test increased Zn concentration in hippocampus increased BDNF in hippocampus	[259]
		1, 5 mg/kg	B6J mice	activation of the channel via motif at TRPC6 anxiolytic and antidepressant effects (OFT)	[260]
	safranal	28 mg/day	128 healthy adults	decrease in negative mood and symptoms related to stress and anxiety	[261]
		50, 200 mg/kg	30 male Wistar rats	no effect in EPM increase in swimming time in FST increase consumption of sweet solution	[262]
	geraniol	20, 40 mg/kg	male ICR mice	alleviation the depression-related behaviors of CUMS-exposed mice regulating IL-1β-related neuroinflammation	[263]
alkaloids	berberine	10, 20 mg/kg	male Wistar rats	increase in serotonin, dopamine, and noradrenaline levels reduction in substance P reduction in lipid peroxide levels decrease immobility time in FST	[264]
		50, 100 mg/kg	male ICR mice	decrease in levels of proinflammatory cytokines in hippocampus	[265]
	linalool	40, 200 mg/kg	male Sprague-Dawley rats	increase in sucrose preference normalization of behavior in OFT decrease motionless time reduction in inflammation in gastrointestinal organs	[266]
	huperzine A	0.05, 0.15 mg/kg	male Sprague-Dawley rats	increase in neurological deficit score increase in cognitive function increase in 5-HT, DA, noradrenaline, and BDNF level in hippocampus and 5-HT and DA in prefrontal cortex	[267]
	galantamine	0.02, 0.2, 2.0 mg/kg	C57BL/6J mice	antidepressant-like effects in FST	[53]

Abbreviations: 5-hydroxytryptamine receptors (5-HT); selective serotonin reuptake inhibitors (SSRIs); superoxide dismutase (SOD); brain-derived neurotrophic factor (BDNF); elevated plus maze (EPM); metachlorophenylpiperazine (MCP); open field test (OFT); tumor necrosis factor α TNF-α; interleukin-1β (IL-1β); subunit nuclear fac-tor kappa B (NF-κβ p56); forced swim test (FST); inducible nitric oxide synthase (iN-OS); nuclear factor kappa B (NF-κβ); CD11b (microglia marker); transient receptor potential canonical 6 channel (TRPC6); 5-hydroxytryptamine 1A receptor (5-HT1A); cAMP-response element-binding protein (CREB); phosphorylated cAMP-response element-binding protein (p-CREB); dopamine (DA); Bcl-2-associated X protein (Bax); B-cell lymphoma-2 (Bcl-2); spared nerve injury (SNI); and canonical 6 channel (TRPC6).

### 4.3. Terpenes and Saponins

Natural bioactive substances such as cyclodepsipeptides and diterpenes are secondary metabolites that exhibit neuroprotective effects by interacting with the GABA-A receptor [268]. Additionally, the nervous system can be enhanced by compounds, such as γ-terpinenes, monoterpenes, and β-pinenes [269].

Hyperforin demonstrated its antidepressant effect when administered to mice daily at a dose of 2.5 and 5 mg/kg i.p. for 3 weeks, when it reversed depressive-like behavior induced by CUMS in the TST, FST, or splash test. It also increased the concentration of Zn and BDNF in the hippocampus [259]. The anxiolytic and antidepressant effect of hyperforin was demonstrated by activating TRPC6 via a specific binding motif, which excited hippocampal neurons in mice and significantly reversed anxiety-like behavior in the OFT. TRPC6 deficiency not only causes anxiety and depression but also reduces the excitability of the CA1 region in the hippocampus [260].

The administration of geraniol for 3 weeks at a dose of 20 and 40 mg/kg had antidepressant effects, as it reversed the effects of CUMS in the sucrose preference test, TST, and FST and reversed the increase in IL-1β induced by CUMS, probably through NF-κB and NLRP3 [263].

Safron from *Crocus sativus* (affron^®^) administered to 128 adult participants for 4 weeks at a dose of 28 mg/day, but not at a dose of 22 mg/day, reduced negative mood and symptoms related to anxiety and stress with no side effects [261]. In another study, 50 p.o. and 200 mg/kg i.p. of affron^®^ showed anxiolytic and antidepressant effects in the FST and sucrose preference test. In the EPM test, no significant effect was observed [262].

## 5. Potential Risks of Herbal Medicine

There has been growing interest from both the scientific community and the public regarding the connection between dietary bioactive compounds and human health. Because many beneficial effects of natural compounds on human health have been described, they are studied very intensively. What must be mentioned are some potential risks, such as low bioavailability, drug interactions, the lack of standardization of natural supplements, and many others. There are several factors influencing the solubility and bioavailability of natural compounds, such as their molecular structure, food matrix effects, transporters, pH variations, and gut microbiota metabolism [270]. The bioavailability of these compounds is critical for understanding their potential health benefits [271]. Recently, this challenge tried to be overcome by structural modifications, colloidal systems, and nanotechnology, aiming to increase the absorption and bioavailability of bioactive compounds [272,273].

Another problem may be drug–herb interactions because natural products may interact with drugs by affecting the biological processes that regulate their metabolism and elimination. Until now, little is found in the literature reporting on the subject “herb–drug interaction,” where most of the reports have been reviews [274]. Even despite the recent efforts to encourage the reporting of adverse drug reactions, the number of reports remains relatively low. Therefore, there is a need for global regulatory harmonization of herbs [275].

## 6. Conclusions

Herbal medicines have been used for thousands of years to treat depression and various diseases. The therapeutic effects of herbal medicines have been proven in the form of monotherapy and complementary therapy for the treatment of mild-to-moderate depression. Many animal models have confirmed the effects (reduction in depressive-like behavior) and neurochemical changes of synthetic antidepressants and plants from herbal medicine. However, these effects have only been demonstrated in a small sample of patients. Despite these facts, it is important that the mechanisms, efficacy, and safety are more clearly demonstrated in both animal and clinical studies.

Conservation, rigorous research grounded in traditional knowledge, stringent quality control, and thorough documentation are crucial in the 21st century to advance the use of herbal medicine.

## Abbreviations

The following abbreviations are used in this manuscript:

5-HT	5-hydroxytryptamine
5-HT1A	5-hydroxytryptamine 1A receptor
5-HT1B	5-hydroxytryptamine 1B receptor
5-HT2	5-hydroxytryptamine receptor 2
ACTH	adrenocorticotropic hormone
AMPA	α-Amino-3-hydroxy-5-methyl-4-isoxazolepropionic acid
Bax	Bcl-2-associated X protein
Bcl-2	B-cell lymphoma-2
BDNF	brain-derived neurotrophic factor
BDNF/TrkB	brain-derived neurotrophic factor/tropomyosin receptor kinase B
CD11b	microglia marker
COX 2	cyclooxygenase-2
CREB	cAMP-response element-binding protein
CRH	corticotrophin-releasing hormone
CXCL	CXC motif chemokine ligand
CXCR2	CXC motif chemokine receptor 2
CYP2A	cytochrome p450 family 2A
CYP2B	cytochrome p450 family 2B
CYP2C11	cytochrome p450, subfamily 2, polypeptide 11
CYP2C9	Cytochrome p450 family 2 subfamily C member 9
CYP1A2	cytochrome p450 family 1A2
CYP2C19	Cytochrome p450 2C19
CYP2C8	cytochrome p450 family 2 subfamily C member 8
CYP3A	cytochrome p450, family 3, subfamily A
D1	dopamine receptor 1
D2	dopamine receptor 2
DA	dopamine
EPM	elevated plus maze
ERK	extracellular-signal-regulated kinase
EtOH	ethyl alcohol
FDA	Food and Drug Administration
FST	forced swim test
GABA	γ-aminobutyric acid
GABA-A	γ-aminobutyric acid type A receptors
GABA-T	γ-aminobutyric acid transaminase
GAD	decarboxylase
GDNF	glial cell line-derived neurotrophic factor
GPCR	G protein-coupled receptor
HPA	hypothalamus–pituitary axis
IDO	indoleam-ine-2,3-dioxygenase
IL	interleukin
IL-1β	interleukin-1β
iNOS	inducible nitric oxide synthase
MAO	monoamine oxidase
MAO A	monoamine oxidase A
MAO B	monoamine oxidase B
MCP	metachlorophenylpiperazine
MDDs	major depressive disorders
MRI	magnetic resonance imaging
NA	noradrenaline
NF-kB	nuclear factor kappa B
NF-κβ p56	subunit nuclear factor kappa B
NGF	nerve growth factor
NLRP1	NLR family pyrin domain containing 1
NLRP3	NLR family pyrin domain containing 3
NMDA	N-methyl-D-aspartate
NO	nitric oxide
NOX	oxides of nitrogen
OFT	open field test
PAF	platelet-activating factor
p-CREB	phosphorylated cAMP-response element-binding protein
PGE2	prostaglandin E2
RNS	reactive nitrogen species
ROS	reactive oxygen species
SIRT1/MAPK	sirtuin 1/mitogen-activated protein kinase
SNI	spared nerve injury
SOD	superoxide dismutase
SSRIs	selective serotonin reuptake inhibitors
TNF-α	tumor necrosis factor α
TRPC6	transient receptor potential canonical 6 channel
WHO	World Health Organization
